# Evaluation of the Potential Risk of Drugs to Induce Hepatotoxicity in Human—Relationships between Hepatic Steatosis Observed in Non-Clinical Toxicity Study and Hepatotoxicity in Humans-

**DOI:** 10.3390/ijms18040810

**Published:** 2017-04-12

**Authors:** Keisuke Goda, Akio Kobayashi, Akemi Takahashi, Tadakazu Takahashi, Kosuke Saito, Keiko Maekawa, Yoshiro Saito, Shoichiro Sugai

**Affiliations:** 1 Toxicology Research Lab., Central Pharmaceutical Research Institute, JAPAN TOBACCO INC., 23 Naganuki Hadano, Kanagawa 257-0024, Japan; akio.kobayashi@jt.com (A.K.); akemi.takahashi@jt.com (A.T.); tadakazu.takahashi@jt.com (T.T.); shoichiro.sugai@jt.com (S.S.); 2 Division of Medicinal Safety Science, National Institute of Health Sciences, Setagaya, Tokyo 158-8501, Japan; saitok2@nihs.go.jp (K.S.); maekawa@nihs.go.jp (K.M.); yoshiro@nihs.go.jp (Y.S.)

**Keywords:** steatosis, drug-induced liver injury, lipidomics, mitochondrial dysfunction

## Abstract

In the development of drugs, we sometimes encounter fatty change of the hepatocytes (steatosis) which is not accompanied by degenerative change in the liver in non-clinical toxicity studies. In this study, we investigated the relationships between fatty change of the hepatocytes noted in non-clinical toxicity studies of compound X, a candidate compound in drug development, and mitochondrial dysfunction in order to estimate the potential risk of the compound to induce drug-induced liver injury (DILI) in humans. We conducted in vivo and in vitro exploratory studies for this purpose. In vivo lipidomics analysis was conducted to investigate the relationships between alteration of the hepatic lipids and mitochondrial dysfunction. In the liver of rats treated with compound X, triglycerides containing long-chain fatty acids, which are the main energy source of the mitochondria, accumulated. Accumulation of these triglycerides was considered to be related to the inhibition of mitochondrial respiration based on the results of in vitro mitochondria toxicity studies. In conclusion, fatty change of the hepatocytes (steatosis) in non-clinical toxicity studies of drug candidates can be regarded as a critical finding for the estimation of their potential risk to induce DILI in humans when the fatty change is induced by mitochondrial dysfunction.

## 1. Introduction

Drug-induced liver injury (DILI) is one of the more serious and frequent drug-related adverse events. This adverse event is a main reason for regulatory action pertaining to drugs, including restrictions in clinical indications and withdrawal from clinical trials or the marketplace [1,2]. Therefore, the estimation of the potential risk of candidate compounds to induce DILI in humans is important to facilitate the development of new drugs, however, the estimation is difficult from the results of standard non-clinical toxicity studies.

Although standard non-clinical toxicity studies have limitations, they sometimes give signals alerting to the risk of DILI in humans. One of these signals is fatty change of the hepatocytes (steatosis). Fatty change of the hepatocytes is frequently observed in non-clinical toxicity studies of drug candidates, especially in rodents. In rodents, fatty change of the hepatocytes is observed even in the control animals as one of the background histopathological findings, and alteration of the nutritional state of the animals, including a decrease in food consumption, leads to the alteration of lipid metabolism in the liver and causes fatty change of the hepatocytes (especially in the periportal hepatocytes of the liver) [3]. On the other hand, there are many drugs causing fatty change of the hepatocytes in non-clinical toxicity studies, especially in rodents and inducing DILI in humans (Table 1). These drugs include amiodarone [4], tamoxifen [5], panadiplon [6], valproic acid [7], amineptin [8], etomoxir [9], and tetracycline [10,11], all of which induce fatty change of the hepatocytes in non-clinical toxicity studies without any degenerative change in the liver. The mechanism of the fatty change is considered to be the inhibition of fatty acid β-oxidation, inhibition of respiration of the mitochondria, or inhibition of the tricarboxylic acid (TCA) cycle (Table 1). One of these drugs, valproic acid, a small branched aliphatic compound, causes fatty change of the hepatocytes in mice and rats [12,13,14]. Valproic acid affects the function of mitochondria by various mechanisms including inhibition of β-oxidation, depletion of free coenzyme A (CoA), uncoupling of the mitochondrial proton gradient, and inhibition of carnitine palmitoyl transferase (CPT) 1. Tetracycline, a broad-spectrum antibiotic, induces macrovesicular fatty change of the hepatocytes in mice [15,16,17]. Some mechanisms considered to be involved in the induction of the fatty change of the hepatocytes induced by tetracycline are prevention of the triglycerides (TGL) export from the liver, and down-regulation of the genes of enzymes/proteins involved in β-oxidation and in the TCA cycle (long-chain acyl CoA dehydrogenase (LCAD), electron transfer flavoprotein (ETF) subunit β, and malate dehydrogenase (MDH)) [18]. LCAD and ETF play important roles for β-oxidation and MDH is a key enzyme in the TCA cycle [19]. Therefore, fatty change of the hepatocytes (steatosis) detected in non-clinical toxicity studies of drug candidates will be a critical finding for the estimation of potential risk of the candidates to induce DILI in humans when the fatty change is induced by mitochondrial dysfunction.

We experienced a drug candidate inducing fatty change of the hepatocytes in rodents (rats) but not in non-rodents (dogs) in standard non-clinical toxicity studies. Therefore, in order to have a better understanding of the relationships between hepatic fatty change observed in non-clinical toxicity studies and DILI in humans, we investigated the mechanism for the fatty change of the hepatocytes using in vivo lipidomics analyses and in vitro mitochondrial toxicity studies for the estimation potential risk of this compound to induce DILI in humans.

## 2. Results

### 2.1. One-Month Repeated Oral Dose Study of Compound X in Rats

The dose levels of 30, 100, and 300 mg/kg were used in this study. The summary of results of the one-month repeated oral dose study in rats is shown in Table 2. Systemic exposure to compound X, as shown by the area under the curve (AUC) of the plasma concentrations of compound X, increased dose-dependently at up to the highest dose level. There were no treatment-related changes at any dose level in the clinical chemistry, necropsy, or histopathology of the liver. The liver weights relative to body weights were increased by 5% at the highest dose level, 300 mg/kg, (*p* < 0.05) when compared with the control group (Table 2). In conclusion, no findings suggestive of hepatotoxicity were seen at up to the highest dose level in the one-month repeated oral dose study in rats.

### 2.2. Three-Month Repeated Oral Dose Study of Compound X in Rats

The dose levels of 100, 300, and 600 mg/kg were used in this study. The summary of results of the three-month repeated oral dose study in rats is shown in Table 3. Systemic exposure to compound X increased dose-dependently at up to the highest dose level. There were treatment-related changes in plasma aspartate aminotransferase (AST), alanine aminotransferase (ALT), and TGL levels. Plasma AST and ALT levels were slightly increased at 600 mg/kg (*p* < 0.01, 1.3-fold and 2.2-fold increases when compared with the control, respectively). The increase in plasma transaminase levels was not accompanied by marked changes in other liver function parameters, including lactate dehydrogenase (LDH) alkaline phosphatase (ALP), and bilirubin (BIL). Plasma TGL levels were decreased at 600 mg/kg (*p* < 0.01) and tended to be decreased at 300 mg/kg. Absolute liver weights were increased at 300 (*p* < 0.05) and 600 mg/kg (*p* < 0.01) and liver weights relative to body weights were increased at 600 mg/kg (*p* < 0.01). Yellowish discoloration in the liver was noted as a macroscopic finding at 600 mg/kg. Although periportal hepatocellular fatty changes were observed in all the animals, including the control group, its severity was increased at 600 mg/kg. This histopathological finding corresponded to the yellowish discoloration seen as a macroscopic finding. There were no degenerative changes in the microscopic examinations. In conclusion, slight increases in plasma ALT and AST levels and hepatocellular fatty changes in the liver were noted at 600 mg/kg, however, there were no degenerative changes in the liver at up to the highest dose level.

### 2.3. Lipidomics for the Liver of Rats Treated with Compound X for One Month

**Figure 1:**
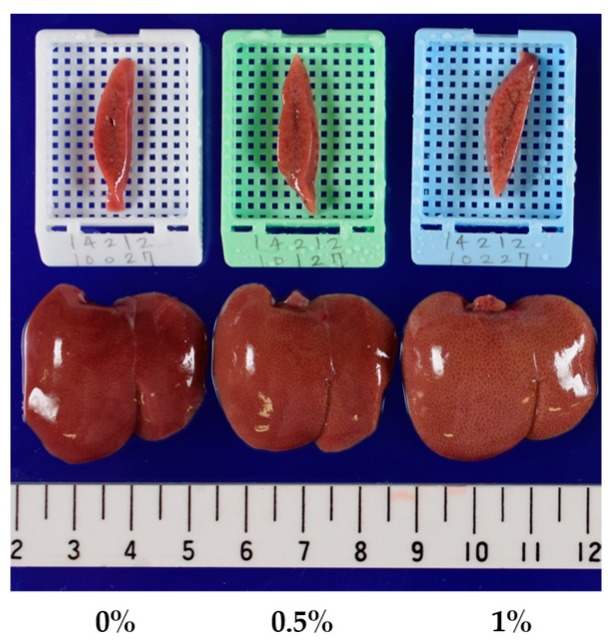
The liver of rats treated with compound X for one month in lipidomics study.

**Figure 2:**
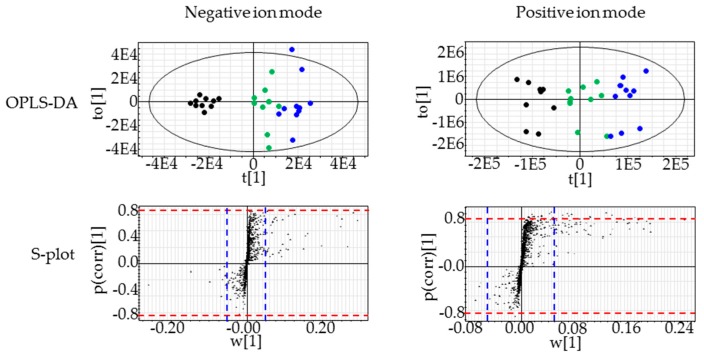
Orthogonal partial least squares-discriminant analysis (OPLS-DA) and s-plot of lipidomics data from the liver collected from rats treated with compound X for one month. Extracted lipid ions that contributed to the discrimination of the control and compound X-treated samples using OPLS-DA. To sort these lipid ions, |w| > 0.05 (blue dotted lines) and |p (corr)| > 0.8 (red dotted lines) in the loading s-plot of the OPLS-DA score, which represent the magnitude of contribution (weight) and reliability (correlation), respectively, were selected as cut-off values. ●: 0%, ●: 0.5%, ●: 1%.

**Figure 3:**
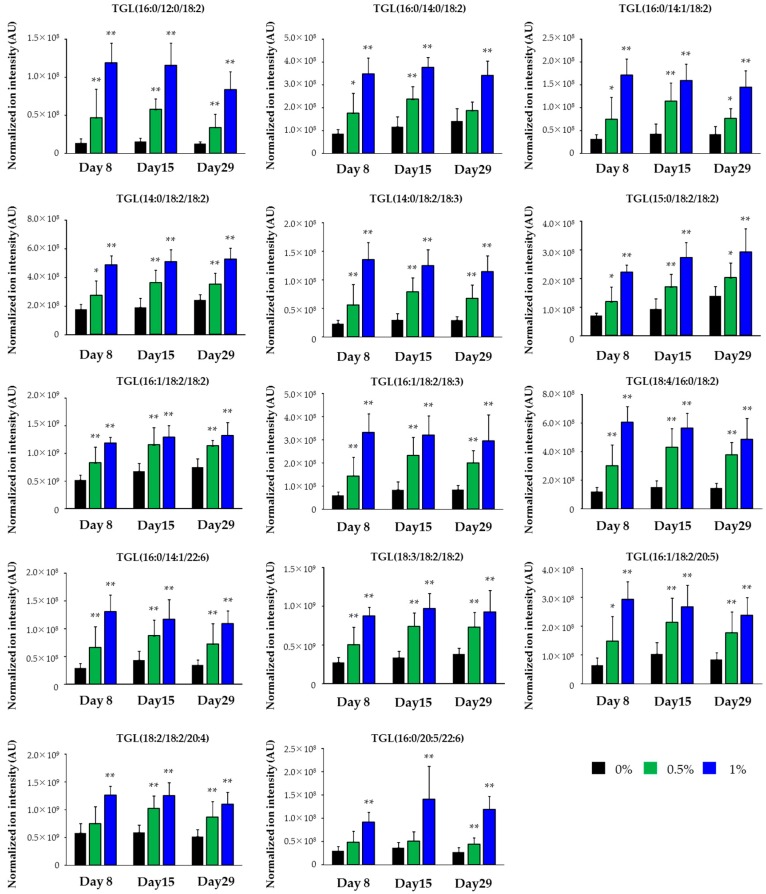
Time-dependent changes in the levels of the characterized triglycerides in the liver of rats treated with compound X. The determined ion intensities of the indicated TGL in positive ion mode were normalized to that of the internal standard (8:0/8:0/18:2 TGL). All data presented are shown as the mean of normalized ion intensity + S.D. (*n* = 10 for all groups). * *p* < 0.05, ** *p* < 0.01 (Dunnett test) AU: Arbitrary unit.

The concentrations of compound X in the basal diet were set at 0.5% and 1% and chemical intake was 380 and 793 mg/kg/day, respectively. Plasma AST and ALT levels were slightly increased at 1% on days 15 and 29 (*p* < 0.01) (1.6-fold and 2.2-fold increases when compared with the control, respectively, on day 29). There were no marked changes in other liver function parameters, including plasma ALP, LDH, and BIL levels. Plasma TGL levels were decreased at 1% on day 29 (*p* < 0.01) and tended to be decreased at 1% on days 8 and 15. Absolute liver weights or liver weights relative to body weights were increased at 1% at all sampling points *(p* < 0.05 or *p* < 0.01). Pathological findings were noted in the liver at 1% and included hepatic yellowish discoloration that histopathologically corresponded to periportal hepatocellular fatty changes (Table 4, Figure 1). Histopathological findings suggestive of hepatotoxicity were not observed at up to the highest dose level in this study as well as in the one- and three-month repeated oral dose studies. Using liver samples collected on day 29, lipidomics analysis provided 1118 and 1299 ion peaks from the liver in negative and positive ion modes, respectively. As shown in Figure 2, the control and compound X-treated rats were clearly discriminated by the orthogonal partial least squares-discriminant analysis (OPLS-DA) plot of the lipidomics data. By screening with |w| > 0.05 and |p (corr)| > 0.8 as threshold values in the s-plot, we obtained 14 lipid ions from the positive ion mode as differential lipid ions between control and compound X treated rat (Table 5). All of the extracted lipids were TGL, and all of them were increased by treatment with compound X. In addition, the dominant composition of these TGL was long-chain fatty acids mainly with 14 to 18 carbon atoms. These increases were observed from day 8 (Figure 3).

### 2.4. Hepatic mRNA Levels of Enzymes Related to Beta-Oxidation in Rats Treated with Compound X

**Figure 4:**
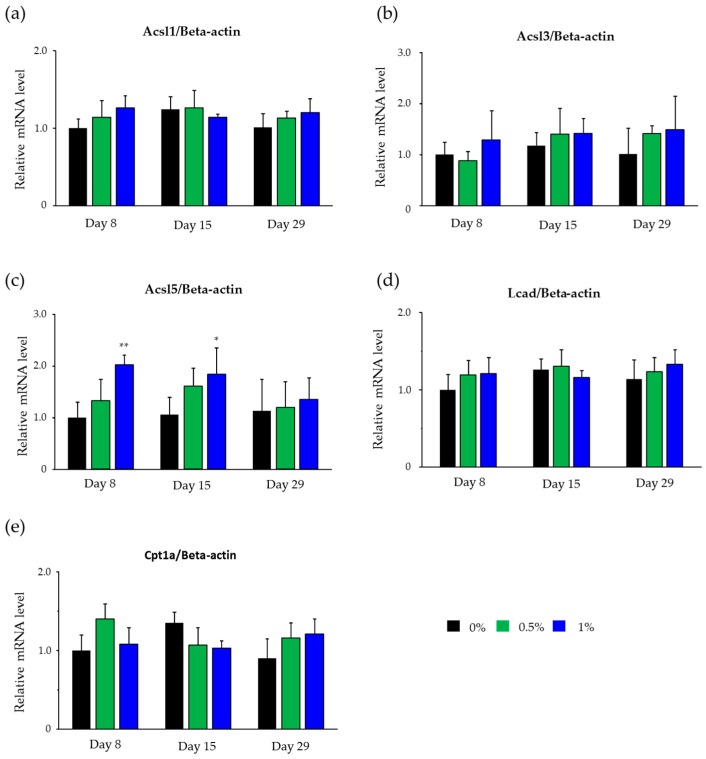
Relative mRNA levels of Acsl1 (**a**), Acsl3 (**b**), Acsl5 (**c**), Lcad (**d**), and Cpt1a (**e**) in the liver of rats treated with compound X for one month. Each bar indicates the mean value (the normalized value corrected by that in the control group on day 8) + S.D. with five determinations. * *p* < 0.05, ** *p* < 0.01 (Dunnett test).

Liver samples collected in the lipidomics study were used in the analyses. Long-chain acyl CoA synthetase (Acsl) 1 and 3 mRNA levels were not changed by the treatment with compound X at any sampling point (Figure 4a,b). Acsl5 mRNA levels were increased dose-dependently on days 8 (*p* < 0.01) and 15 (*p <* 0.05), however, these were not changed on day 29 by the treatment with compound X (Figure 4c). Long-chain acyl CoA dehydrogenase (Lcad) and carnitine palmitoyltransferase 1a (Cpt1a) mRNA levels were not changed by the treatment with compound X at any sampling point (Figure 4d,e).

### 2.5. Effects of Compound X on Mitochondrial Oxygen Consumption in Rat Hepatocyte

**Figure 5:**
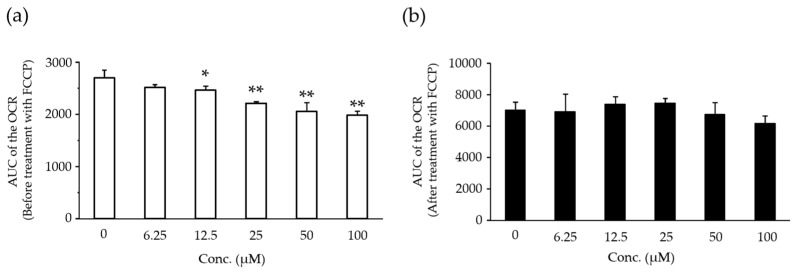
Area under the curve (AUC) of the oxygen consumption rate (OCR) before treatment with carbonyl cyanide-*p*-trifluoromethoxyphenylhydrazone (FCCP) (**a**) and after treatment with FCCP (**b**) in the rat hepatocytes treated with compound X. Each bar indicates the mean value + S.D. with four determinations. * *p* < 0.05, ** *p* < 0.01 (Dunnett test).

The AUC of the oxygen consumption rate (OCR) before treatment with carbonyl cyanide-*p*-trifluoromethoxyphenylhydrazone (FCCP) was decreased dose-dependently at 12.5 µM and above (*p* < 0.05 or *p* < 0.01) (Figure 5a). The AUC of the OCR after treatment with FCCP did not change at any dose level (Figure 5b).

### 2.6. Effects of Compound X on Mitochondrial Oxygen Consumption and Apoptotic Action of Compound X in Human Hepatocytes

**Figure 6:**
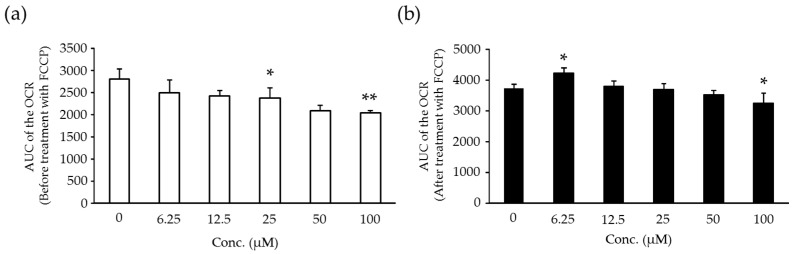
AUC of the OCR before treatment with FCCP (**a**) and after treatment with FCCP (**b**) in the human hepatocytes treated with compound X. Each bar indicates the mean value + S.D. with four determinations. * *p* < 0.05, ** *p* < 0.01 (Dunnett test).

**Figure 7:**
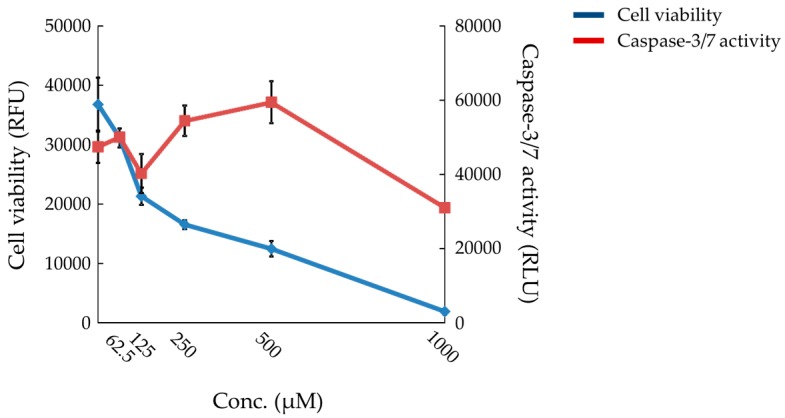
Cell viability and caspase-3/7 activity in the human hepatocytes treated with compound X. Each point indicates the mean value ± S.D. with four determinations. RFU: relative fluorescence unit. RLU: relative luminescence unit.

The AUC of the OCR before treatment with FCCP was decreased dose-dependently at 25 µM and above (*p* < 0.05 or *p* < 0.01) (Figure 6a). The AUC of the OCR after treatment with FCCP was decreased slightly at 100 μM (*p* < 0.05) (Figure 6b). The cell viability was markedly decreased after treatment with compound X at 125 μM and above (Figure 7). Caspase-3/7 activity was increased at 250 and 500 μM (Figure 7).

## 3. Discussion

In the development of drugs, we sometimes encounter fatty change of the hepatocytes which is not accompanied by any degenerative changes in the liver in non-clinical toxicity studies, especially in rodents. There are many drugs that cause hepatic fatty change in rodents, and some of them have the potential to induce DILI in humans through mitochondrial toxicity [4,5,6,7,8,9,10,11]. Mitochondria play a crucial role not only in cell death of the hepatocytes, which leads to DILI in humans [20], but also in the metabolism of fatty acids as a major energy source [21]. Therefore, it is important to investigate the relationships between fatty change of the hepatocytes and the risk of DILI in humans in terms of the effects on mitochondrial function when fatty change is observed in standard non-clinical toxicity studies of drug candidates. We encountered fatty changes in toxicity studies in rats for compound X, a candidate compound in drug development, and conducted some in vivo and in vitro exploratory studies for the compound in order to investigate the mechanism of the fatty change and to estimate potential risk of DILI in humans.

In the one-month repeated dose study in rats, although liver weights were slightly increased at the highest dose level (300 mg/kg), there were no treatment-related histopathological findings at up to the highest dose level (Table 2). In the three-month repeated dose study in rats, plasma AST and ALT levels were slightly increased at the highest dose level (600 mg/kg). The increase in plasma transaminase levels was not accompanied by changes in other liver function parameters, including ALP, BIL, and LDH. A slight increase in plasma transaminase (ALT and AST) level, which is not accompanied by changes in other liver function parameters or degenerative changes in the liver, is sometimes related to the pharmacology of the compounds or is an adaptive change to compounds and is not related to toxicity from the compounds [22]. Thus, the slight increase in transaminase level noted in the three-month repeated dose study was considered not to be of toxicological significance. Plasma TGL levels were decreased at 300 mg/kg and above. Liver weights were increased at 300 mg/kg and above. Fatty change of the hepatocytes was noted at 600 mg/kg, however, there were no degenerative changes in the liver at up to the highest dose level. We also conducted one- and three-month repeated dose studies for compound X in dogs with dose levels giving systemic exposure to compound X comparable to that in rats. In these dog studies, there were neither fatty change of the liver nor findings suggestive of hepatotoxicity, including alteration of liver function parameters at up to the highest dose levels [23,24]. Hepatic fatty change was noted in rats but not in dogs after treatment with compound X, although systemic exposure to compound X was similar between rats and dogs in these repeated dose studies. This result indicates that rats are more sensitive to drug-induced fatty change (steatosis) than dogs. Although the etiology of species difference in sensitivity to drug-induced steatosis between these two animal species is not known, higher base levels of fat in the liver in rats may contribute to higher sensitivity to drug-induced-steatosis in rats; the control rats have fat in the periportal hepatocytes in the liver, as observed in our present studies. Decreased plasma TGL levels were noted at dose levels equal to or lower than those where hepatocellular fatty change was noted in the three-month repeated dose study in rats. In dogs, plasma TGL levels did not change after treatment with compound X. Although the etiology of the TGL-lowering effect of compound X is not known, this change was considered to be related to the fatty change in the liver.

**Figure 8:**
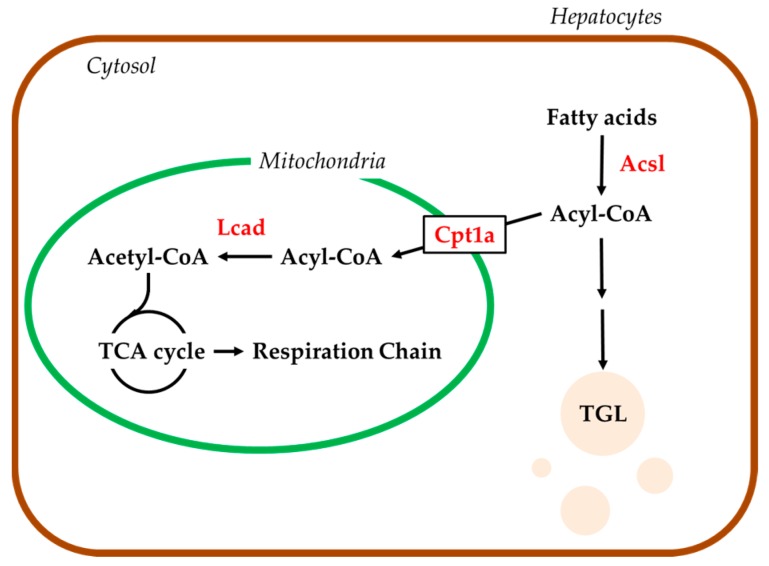
Metabolic pathway of the fatty acid into TGL storage and β-oxidation in hepatocytes.

In order to investigate the effects of compound X on lipid metabolism in rats, we conducted lipidomics analysis in rats treated with compound X for up to one month. The lipidomics revealed that TGL that accumulated in the hepatocytes after treatment with compound X consisted of long-chain fatty acids mainly with 14 to 18 carbon atoms, which are known to be the main energy source of mitochondria (Table 5). In order to investigate whether compound X inhibits fatty acid β-oxidation, we measured hepatic mRNA levels of Acsl1, 3, and 5, Lcad, and Cpt1a, all of which play important role for the β-oxidation of long-chain fatty acids (from 12 to 20 carbon atoms), in the lipidomics study (Figure 8). The mRNA levels of Acsl1 and 3, Lcad, and Cpt1a did not change after treatment with compound X at any sampling point (Figure 4), indicating that compound X does not directly inhibit fatty acid β-oxidation. On the other hand, Acsl5 mRNA level was increased by the treatment with compound X at the early phase of the dosing period. Each of Acsl isoforms is known to have an individual function for fatty acid metabolism [25,26,27]. In particular, Acsl5 is considered to be important as a branch point in directing fatty acids into TGL storage or β-oxidation [28]. In addition, it has been reported that overexpression of Acsl5 increases fatty acid incorporation into TGL in the hepatocyte cell line [29]. These results indicated that fatty acids, which could not be used in the mitochondria due to effects on mitochondrial function other than inhibition of β-oxidation, were incorporated into TGL by Ascl5. In order to investigate whether compound X induces mitochondrial dysfunction, we measured basal mitochondrial function and maximum mitochondrial function by determining the AUC of the OCR before treatment with FCCP and the AUC of the OCR after treatment with FCCP, respectively, after treating rat primary hepatocytes with compound X. The basal mitochondrial function was decreased dose-dependently by treatment with compound X (Figure 5a). The maximum mitochondrial function was not changed by treatment with compound X (Figure 5b). From the results of these mechanistic studies, the fatty acids could not be used for mitochondrial respiration due to mitochondrial toxicity caused by compound X. Therefore, compound X is considered to induce fatty change of the liver in rats through mitochondrial toxicity.

Finally, we estimated the potential risk of compound X to induce DILI in humans using in vitro combination assays of mitochondrial dysfunction and apoptosis as endpoints, which we have previously reported as a useful tool for estimation of risk of DILI [30]. Compound X induced both mitochondrial dysfunction and apoptosis in human primary hepatocytes in this assay (Figure 6 and Figure 7). The positive results on these two endpoints strongly indicate that compound X has the potential to induce DILI in humans.

In conclusion, fatty change of the hepatocytes (steatosis) detected in non-clinical toxicity studies of drug candidates in rodents can be regarded as a critical finding for the estimation of the potential risk of the candidates to induce DILI in humans when the fatty change is induced by mitochondrial dysfunction. Based on the results of the exploratory studies conducted for compound X, lipidomics and mitochondrial toxicity studies are useful to estimate the potential risk of DILI in humans for compounds which cause fatty change in the standard non-clinical toxicity studies in rodents.

## 4. Materials and Methods

### 4.1. Materials

The Compound X, a candidate compound for drug development, was synthesized in Central Pharmaceutical Research Institute, Japan Tobacco Inc. (Osaka, Japan). All other reagents were obtained commercially and were the highest grade available.

### 4.2. Animals

Five-week-old male Crl:CD (SD) rats were purchased from Charles River Japan Inc. (Kanagawa, Japan). The animals were housed individually in wire-mesh cages kept in an air-conditioned room with a 12-h light-dark cycle (lighting from 7:00 a.m. to 7:00 p.m.) at a temperature of 23 ± 1 °C, a relative humidity of 55% ± 5%, and a ventilation rate of about 15 times per hour. The rats were quarantined for 1 week and were allowed free access to a commercial powder diet (CRF-1, Oriental Yeast Co., Ltd., Tokyo, Japan) ad libitum. Tap water was available for drinking ad libitum.

All animal experimental procedures were approved by the Institutional Animal Care and Use Committee of the Toxicology Research Laboratories, Central Pharmaceutical Research Institute, Japan Tobacco Inc. This study was conducted in accordance with the Japanese Law for the Humane Treatment and Management of Animals (Law No. 105, as revised in 2013, issued in 1 October 1973).

### 4.3. One and Three-Month Repeated Oral Dose Studies

#### 4.3.1. Dosing

Compound X was suspended in 0.5% methylcellulose (MC, Shin-etsu Chemical Co., Ltd., Tokyo, Japan) aqueous solution. The rats were given compound X once daily for 28 and 91 days in one- and three-month repeated oral dose studies, respectively. Dose levels used in the one-month study were 30, 100, and 300 mg/kg. In the three-month study, dose levels of 100, 300, and 600 mg/kg were selected because there were no toxicological changes in the one-month repeated oral dose study.

#### 4.3.2. Sampling of Blood Samples

The rats were fasted overnight on the last day of the dosing periods, and blood and liver samples were collected the next day. The abdomens of rats were opened under isoflurane anesthesia, and blood samples were collected from the abdominal aorta. The blood samples were transferred into tubes containing heparin as an anticoagulant, and were centrifuged at 1750× *g* for 30 min at 4 °C to obtain plasma.

#### 4.3.3. Measurements of Plasma Liver Function Parameters and TGL Levels

The measurements of plasma liver function parameters and TGL levels were conducted by an automated analyzer (TBA-120FR, TOSHIBA Corporation, Tokyo, Japan) using standard reagents for the clinical chemistry (Wako Pure Chemicals, Tokyo, Japan).

#### 4.3.4. Histopathology

The livers were removed, weighed, and preserved in neutral buffered formalin for histopathological examination. The liver slices were embedded in paraffin. Sectioning and hematoxylin-eosin staining was performed according to routine histological procedures.

#### 4.3.5. Toxicokinetic Analyses

Blood samples were collected from the animals in the one- and three-month repeated dose studies for measurements of the concentrations of compound X in plasma. The concentration was measured by liquid chromatography-tandem mass spectrometry (LC/MS/MS) using the internal standard method.

### 4.4. Lipidomics Study

#### 4.4.1. Dosing

Compound X was mixed with powder basal diet (CRF-1) at 0.5% and 1%, and rats were given the admixture diet for 7, 14, or 28 days. Chemical intakes of compound X at 0.5% and 1% were 380 and 793 mg/kg/day, respectively. These dose levels and dosing methods were considered to be enough to detect fatty change of the hepatocyte induced by compound X for up to one-month based on the results of a preliminary study (data not shown).

#### 4.4.2. Sampling of Blood and Liver

Blood and liver were collected on the next day (days 8, 15, and 29) of the last day of each dosing period.

The blood samples were transferred into tubes containing ethylenediaminetetraacetic acid-2 potassium (EDTA-2K) as an anticoagulant and were centrifuged at 1750× *g* for 30 min at room temperature to obtain plasma for lipidomics. The samples were frozen with liquid nitrogen and stored at −80 °C until use. The livers for lipidomics were frozen with liquid nitrogen stored at −80 °C until use.

#### 4.4.3. Lipidomics

The procedure of the lipidomics was described previously [31].

For liver lipid extraction, 20 mg/mL homogenate were prepared with methanol. Liver lipids were extracted by mixing 100 µL of liver homogenate with 100 µL of methanol:chloroform (1:1) containing 2 µM PE(12:0/12:0) and 0.5 µM TG(8:0/8:0/18:2) as internal standards. After mixing, both homogenates were centrifuged at 15,000× *g* for 4 min to precipitate debris. The supernatants were collected, filtered, and stored at a temperature of −80 °C until use.

Measurements of the lipid content were performed with LC/MS measurement as previously described [32]. For the lipid ion quantification, the raw full MS data obtained by LC/MS were processed using the 2DICAL software (Mitsui Knowledge Industry, Tokyo, Japan), which allowed the alignment of the detected ion peaks of each biomolecule obtained at a specific *m*/*z* with the column retention time (RT). The main parameters of the 2DICAL software were set as described previously [33]. For samples with missing values for a lipid ion, 50,000 (negative ion mode) or 500,000 (positive ion mode) was applied. The intensity of each extracted ion peak was normalized to those of the internal standard (PE(12:0/12:0) for negative ion mode and TG(8:0/8:0/18:2) for positive ion mode). The values of the relative standard deviation of the internal standard (PE(12:0/12:0) and TG(8:0/8:0/18:2)) were monitored for experimental quality control throughout the extraction, measurement, and data processing and were 10.0% and 10.9% for plasma and 7.3% and 7.1% for the liver, respectively.

#### 4.4.4. OPLS-DA and Lipid Identification

The control and compound X-treated data sets for the intensities of the extracted lipid ions from rat plasma and liver were loaded into SIMCA-P+ 12 (Umetrics, Umea, Sweden), pareto-scaled, and analyzed using OPLS-DA to extract lipid ions that contributed to the discrimination of the control and compound X-treated samples. To sort these lipid ions, |w| > 0.05 and |p (corr)| > 0.8 in the loading s-plot of the OPLS-DA score, which represent the magnitude of contribution (weight) and reliability (correlation), respectively, were selected as cut-off values. Subsequently, the lipid ions identified as discriminant factors were subjected to identification of lipid molecules as described previously [30,31].

#### 4.4.5. Real-Time PCR

Aliquots of the tissue samples were homogenized by Tissue Lyser (QIAGEN, Hilden, Germany) and the total RNA was extracted using RNeasy Mini kit (QIAGEN) according to the manufacturer’s instructions. Next, 2.0 μg of the isolated total RNA was used to synthesize cDNA with SuperScript VILO Mastermix (Invitrogen, Carlsbad, CA, USA) according to the manufacturer’s instructions. The synthesized cDNA solutions were diluted 5-fold by Tris-EDTA (TE) buffer (pH 8.0, NIPPON GENE Co., Ltd., Tokyo, Japan). Before the measurements, the cDNA solutions were further diluted 10-fold with MILLI-Q water (Millipore Corporation, Darmstadt, Germany) and were used for TaqMan probe-based semi quantitative-real time PCR. The mRNA levels of Acsl1, 3, and 5, Lcad, Cpt1a, and β-actin were measured in duplicate on a 7300 Real-Time PCR System (Applied Biosystems, Waltham, MA, USA) using TaqMan Gene Expression Master Mix (Applied Biosystems) according to the manufacturer’s instructions. The data analysis was performed by a calibration curve method using SDS software (Applied Biosystems) and the results were normalized to actb expressions.

The following primer and TaqMan probe mixtures were obtained from Applied Biosystems: Acsl1 (Rn00563137_m1), Acsl3 (Rn00589037_m1), Acsl5 (Rn00586013_m1), Lcad (Rn00563121_m1), Cpt1a (Rn00580702_m1), and Beta-actin (Rn00667869_m1).

### 4.5. In Vitro Studies

#### 4.5.1. Rat Hepatocytes

Commercial cryopreserved rat primary hepatocytes obtained from Gibco™ (Invitrogen, Carlsbad, CA, USA) were used.

Cryopreserved hepatocytes were thawed in a 37 °C-water bath and transferred into plating medium (William’s medium E (Invitrogen) containing Hepatocytes Plating Supplement Pack (Invitrogen)). The cell suspension was centrifuged at 100× *g* for 10 min at room temperature and the supernatant was removed. The pelleted cells were suspended in plating medium, part of the cell suspension was stained with 0.4% Trypan blue (Invitrogen), and the number of cells was counted microscopically using a cell counting chamber. Aliquots of the hepatocytes suspension (6 × 10^4^ cells/100 μL/well) were added to the collagen-coated culture plate (percentage of live cells was more than 80%). A 96-well white cell culture plate (clear bottom) was used for the measurements of cell viability and caspase-3/7 activity and a 24-well cell culture plate designed for XF24 Extracellular Flux Analyzer (Seahorse bioscience, Inc., North Billerica, MA, USA) was used for the measurements of OCR. The medium was replaced with 200 µL incubation medium (William’s medium E containing Hepatocytes Maintenance Supplement Pack (Invitrogen)) after 4 to 6 h of plating. Stationary culturing was carried out at 37 °C in a humidified (100%) atmosphere containing 5% CO_2_. In all the assays, four wells were used per dose.

#### 4.5.2. Human Hepatocytes

Commercial cryopreserved human primary hepatocytes obtained from Gibco™ (Invitrogen) were used.

Cryopreserved hepatocytes were thawed in a 37 °C-water bath and transferred into Cryopreserved Hepatocytes Recovery Medium (Invitrogen). The cell suspension was centrifuged at 100× *g* for 10 min at room temperature and the supernatant was removed. The pelleted cells were suspended in plating medium (William’s medium E (Invitrogen) containing Hepatocytes Plating Supplement Pack (Invitrogen)), part of the cell suspension was stained with 0.4% Trypan blue (Invitrogen), and the number of cells was counted microscopically using a cell counting chamber. Aliquots of the hepatocytes suspension (6 × 10^4^ cells/100 μL/well) were added to the collagen-coated culture plate (percentage of live cells was more than 90%). A 96-well white cell culture plate (clear bottom) was used for the measurements of cell viability and caspase-3/7 activity and a 24 well cell culture plate designed for XF24 Extracellular Flux Analyzer (Seahorse bioscience, Inc.) was used for the measurements of OCR. The medium was replaced with 200 µL incubation medium (William’s medium E containing Hepatocytes Maintenance Supplement Pack (Invitrogen)) after 4 to 6 h of plating. Stationary culturing was carried out at 37 °C in a humidified (100%) atmosphere containing 5% CO_2_. In all the assays, four wells were used per dose.

#### 4.5.3. Measurements of Cell Viability and Caspase-3/7 Activity

Treatment with compound X was conducted on the day following cell plating, and the hepatocytes were treated with compound for 24 h. The formulations of compound X were prepared with incubation medium (including 1% dimethyl sulfoxide (DMSO)). The activities of live cell protease and caspase-3/7 were measured as indices of cell viability and apoptosis, respectively, using the commercial ApoLive-Glo™ Multiplex Assay (Promega Corporation, Fitchburg, WI, USA) according to the manufacturer’s instructions. A microplate reader (Infinite M200Pro, Tecan Group Ltd. (Zürich, Switzerland)) and analysis software (Magellan V7.2, Tecan Group Ltd.) were used for the measurements of fluorescence and luminescence. The cell viability and caspase-3/7 activity were represented as relative fluorescence units (RFU) and relative luminescence units (RLU), respectively.

#### 4.5.4. Measurements of OCR

The procedure of the measurements of OCR was described previously [30].

An assay was conducted on the next day of cell plating. The assay medium (Dulbecco’s Modified Eagle’s Medium (Sigma-Aldrich, without glucose, l-glutamine, phenol red, sodium pyruvate, and sodium bicarbonate, powder) containing 11 mM glucose, 4 mM sodium pyruvate, and Glutamax (Invitrogen)) was prepared and pH was adjusted to 7.4 at 37 °C by adding NaOH immediately before use. The formulations of compounds were prepared with the assay medium (including 10% DMSO (final concentration: 1%)). The OCR was measured using the XF24 Extracellular Flux Analyzer (Fluxanalyzer, Seahorse bioscience, Inc.). The compound X or vehicle solution and the FCCP solution were loaded automatically in the XF24 Extracellular Flux Analyzer.

### 4.6. Statistical Analysis

All numerical data are shown as mean ± or + standard deviation. The differences in the data were determined using one-way analysis of variance (ANOVA), followed by pairwise comparisons (Dunnett test). The levels of significance were set at 5% and 1% (two-tailed).

## Figures and Tables

**Table 1:** The relationship between clinical hepatotoxicity and steatosis observed in non-clinical toxicity studies.

Drug	Risk of DILI in Humans	Effects on Mitochondria	Animal Species with Steatosis in Non-Clinical Studies
Amiodarone	+	Inhibition of β-oxidation (BO) and respiratory chain (RC)	Mouse and rat
Tamoxifen	+	Inhibition of BO and RC	Rat
Panadiplon *	+	Inhibition of BO	Monkey and rabbit
Valproic acid	+	Inhibition of BO and RC	Mouse and rat
Amineptin	+	Inhibition of BO	Mouse
Etomoxir	+	Inhibition of BO	Rat
Tetracycline	+	Inhibition of BO and tricarboxylic acid cycle (TC)	Mouse and rat

DILI: drug-induced liver injury, *: withdrawn, +: positive.

**Table 2:** Summary results of the one-month repeated oral dose of compound X study in rats.

Dose (mg/kg/day)	0	30	100	300
Number of Animals	12	12	12	12
Systemic exposure; AUC_0–24_ (μmol·h/L) (Day 27)	NA	1.5	9.2	54.1
Clinical Chemistry				
AST (U/L)	66.3 ± 6.5	63.1 ± 6.6	66.5 ± 8.9	64.0 ± 5.4
ALT (U/L)	28.9 ± 5.7	27.3 ± 5.5	29.8 ± 6.3	30.5 ± 4.8
TGL (mg/dL)	67.9 ± 25.2	72.6 ± 26.4	60.8 ± 17.6	58.3 ± 16.7
Liver Weights				
Absolute (g)	10.328 ± 1.329	10.915 ± 0.741	10.121 ± 1.056	11.213 ± 1.233
Relative (g/100 g BW)	2.868 ± 0.193	2.857 ± 0.128	2.792 ± 0.137	3.018 ± 0.126 *
Macroscopic findings				
Liver	-	-	-	-
Microscopic findings				
Liver	-	-	-	-

AUC: area under the curve, NA: Not applicable, BW: body weight, -: No finding, AST: Aspartate aminotransferase, ALT: Alanine aminotransferase, TGL: Triglycerides. *: *p* < 0.05 (Dunnett test).

**Table 3:** Summary results of the three-month repeated oral dose study of compound X in rats.

Dose (mg/kg/day)	0	100	300	600
Number of Animals	15	15	15	15
Systemic exposure; AUC_0–24_ (μmol·h/L) (Day 90)	NA	9.2	50.5	101.1
Clinical Chemistry				
AST (U/L)	69.1 ± 11.5	65.3 ± 8.0	61.3 ± 8.0	92.9 ± 18.5 **
ALT (U/L)	31.9 ± 6.8	30.5 ± 6.0	29.8 ± 4.9	71.3 ±19.4 **
LDH (U/L)	55.9 ± 10.8	59.9 ± 23.3	54.1 ± 12.0	69.3 ± 19.2
ALP (U/L)	194.2 ± 36.0	196.9 ± 21.2	238.5 ± 23.0 **	249.6 ± 38.9 **
BIL (mg/dL)	0.051 ± 0.007	0.051 ± 0.007	0.050 ± 0.007	0.049 ± 0.010
TGL (mg/dL)	83.5 ± 34.3	80.3 ± 26.8	70.3 ± 21.5	46.1 ± 12.2 **
Macroscopic Findings of the liver				
Yellowish discoloration	- : 15	- : 15	- : 14	- : 4
			P : 1	P : 11
Liver Weights				
Absolute (g)	14.327 ± 1.497	15.560 ± 2.024	16.021 ± 2.106 *	17.622 ± 1.555 **
Relative (g/100 g BW)	2.569 ± 0.169	2.572 ± 0.173	2.673 ± 0.128	2.988 ± 0.161 **
Microscopic findings of the liver				
Fatty change, hepatocyte, periportal	± : 6	± : 5	± : 4	2+ : 7
	+ : 4	+ : 8	+ : 11	3+ : 8
	2+ : 5	2+ : 2		

NA: Not applicable, BW: body weight, AST: Aspartate aminotransferase, ALT: Alanine aminotransferase, LDH: Lactate dehydrogenase, ALP: Alkaline phosphatase, BIL: Bilirubin, TGL: Triglycerides, -: Finding absent, ±: very slight, +: slight, 2+: moderate, 3+: severe, P: Finding present. *: *p* < 0.05, **: *p* < 0.01 (Dunnett test).

**Table 4:** Summary results of the one-month lipidomics study of compound X in rats.

Dose (mg/kg/day)		0%	0.5%	1%
Number of animals/sampling point		10	10	10
Chemical Intake (mg/kg/day)		NA	380	793
Clinical Chemistry				
AST (U/L)	Day 8	64.0 ± 6.1	63.5 ± 5.6	67.1 ± 8.7
	Day 15	54.8 ± 6.0	53.8 ± 7.4	67.2 ± 12.4 **
	Day 29	47.9 ± 7.8	53.3 ± 3.3	75.3 ± 15.0 **
ALT (U/L)	Day 8	15.4 ± 2.1	16.4 ± 2.8	17.1 ± 2.4
	Day 15	14.9 ± 2.2	15.0 ± 3.5	22.3 ± 7.9 **
	Day 29	15.2 ± 3.1	17.5 ± 4.0	33.6 ± 15.3 **
LDH (U/L)	Day 8	58.2 ± 15.8	58.9 ± 23.9	65.4 ± 21.5
	Day 15	38.6 ± 10.1	43.8 ± 5.6	53.4 ± 10.1 **
	Day 29	40.7 ± 15.9	41.8 ± 8.0	48.8 ± 11.4
ALP (U/L)	Day 8	696.2 ± 151.7	742.3 ± 106.9	611.1 ± 97.3
	Day 15	583.7 ± 108.2	583.1 ± 93.4	579.2 ± 68.2
	Day 29	373.0 ± 51.9	466.0 ± 79.9 *	452.3 ± 79.4 *
BIL (mg/dL)	Day 8	0.023 ± 0.005	0.020 ± 0.005	0.018 ± 0.006
	Day 15	0.026 ± 0.007	0.025 ± 0.005	0.021 ± 0.006
	Day 29	0.024 ± 0.007	0.029 ± 0.006	0.027 ± 0.009
TGL (mg/dL)	Day 8	33.5 ± 17.4	28.1 ± 11.0	24.9 ± 10.5
	Day 15	30.4 ± 10.9	27.0 ± 19.4	19.0 ± 5.3
	Day 29	42.3 ± 18.0	34.9 ± 15.8	18.3 ± 8.0 **
Macroscopic Findings of the liver				
Yellowish discoloration	Day 8	- : 10	- : 10	- : 10
	Day 15	- : 10	- : 10	P : 10
	Day 29	- : 10	- : 9	P : 10
			P : 1	
Liver Weights				
Day 8	Absolute (g)	7.642 ± 0.645	7.678 ± 0.463	8.367 ± 0.528 *
	Relative (g/100 g BW)	3.176 ± 0.237	3.260 ± 0.144	3.531 ± 0.177 **
Day 15	Absolute (g)	8.710 ± 0.484	8.730 ± 0.644	9.113 ± 0.641
	Relative (g/100 g BW)	2.967 ± 0.119	2.999 ± 0.141	3.269 ± 0.159 **
Day 29	Absolute (g)	10.215 ± 1.449	10.555 ± 1.243	10.875 ± 0.940
	Relative (g/100 g BW)	2.812 ± 0.194	2.760 ± 0.149	2.999 ± 0.100 *
Microscopic findings of the liver				
Fatty change, hepatocyte, periportal	Day 8	- : 6	- : 1	± : 5
		± : 4	± : 9	+ : 5
	Day 15	- : 1	- : 2	+ : 2
		± : 9	± : 8	2+ : 8
	Day 29	± : 4	- : 2	+ : 1
		+ : 6	± : 5	2+ : 8
			+ : 3	3+ : 1

NA: Not applicable, BW: body weight, AST: Aspartate aminotransferase, ALT: Alanine aminotransferase, LDH: Lactate dehydrogenase, ALP: Alkaline phosphatase, BIL: Bilirubin, TGL: Triglycerides, -: Finding absent, ±: very slight, +: slight, 2+: moderate, 3+: severe, P: Finding present. *: *p* < 0.05, **: *p* < 0.01 (Dunnett test).

**Table 5:** Characterized hepatic lipids altered by treatment with compound X.

Class	Lipid	Polarity	Response
TGL	TGL(16:0/12:0/18:2)	Posi	Increase
TGL	TGL(16:0/14:0/18:2)	Posi	Increase
TGL	TGL(16:0/14:1/18:2)	Posi	Increase
TGL	TGL(14:0/18:2/18:2)	Posi	Increase
TGL	TGL(14:0/18:2/18:3)	Posi	Increase
TGL	TGL(15:0/18:2/18:2)	Posi	Increase
TGL	TGL(16:1/18:2/18:2)	Posi	Increase
TGL	TGL(16:1/18:2/18:3)	Posi	Increase
TGL	TGL(18:4/16:0/18:2)	Posi	Increase
TGL	TGL(16:0/14:1/22:6)	Posi	Increase
TGL	TGL(18:3/18:2/18:2)	Posi	Increase
TGL	TGL(16:1/18:2/20:5)	Posi	Increase
TGL	TGL(18:2/18:2/20:4)	Posi	Increase
TGL	TGL(16:0/20:5/22:6)	Posi	Increase

Posi: Positive ion mode.
